# Sodium Pumps Mediate Activity-Dependent Changes in Mammalian Motor Networks

**DOI:** 10.1523/JNEUROSCI.2005-16.2016

**Published:** 2017-01-25

**Authors:** Laurence D. Picton, Filipe Nascimento, Matthew J. Broadhead, Keith T. Sillar, Gareth B. Miles

**Affiliations:** School of Psychology and Neuroscience, University of St Andrews, St Andrews KY16 9JP, United Kingdom

**Keywords:** central pattern generator, locomotion, mouse, Na^+^/K^+^-ATPase, sodium pump, spinal cord

## Abstract

Ubiquitously expressed sodium pumps are best known for maintaining the ionic gradients and resting membrane potential required for generating action potentials. However, activity- and state-dependent changes in pump activity can also influence neuronal firing and regulate rhythmic network output. Here we demonstrate that changes in sodium pump activity regulate locomotor networks in the spinal cord of neonatal mice. The sodium pump inhibitor, ouabain, increased the frequency and decreased the amplitude of drug-induced locomotor bursting, effects that were dependent on the presence of the neuromodulator dopamine. Conversely, activating the pump with the sodium ionophore monensin decreased burst frequency. When more “natural” locomotor output was evoked using dorsal-root stimulation, ouabain increased burst frequency and extended locomotor episode duration, whereas monensin slowed and shortened episodes. Decreasing the time between dorsal-root stimulation, and therefore interepisode interval, also shortened and slowed activity, suggesting that pump activity encodes information about past network output and contributes to feedforward control of subsequent locomotor bouts. Using whole-cell patch-clamp recordings from spinal motoneurons and interneurons, we describe a long-duration (∼60 s), activity-dependent, TTX- and ouabain-sensitive, hyperpolarization (∼5 mV), which is mediated by spike-dependent increases in pump activity. The duration of this dynamic pump potential is enhanced by dopamine. Our results therefore reveal sodium pumps as dynamic regulators of mammalian spinal motor networks that can also be affected by neuromodulatory systems. Given the involvement of sodium pumps in movement disorders, such as amyotrophic lateral sclerosis and rapid-onset dystonia parkinsonism, knowledge of their contribution to motor network regulation also has considerable clinical importance.

**SIGNIFICANCE STATEMENT** The sodium pump is ubiquitously expressed and responsible for at least half of total brain energy consumption. The pumps maintain ionic gradients and the resting membrane potential of neurons, but increasing evidence suggests that activity- and state-dependent changes in pump activity also influence neuronal firing. Here we demonstrate that changes in sodium pump activity regulate locomotor output in the spinal cord of neonatal mice. We describe a sodium pump-mediated afterhyperpolarization in spinal neurons, mediated by spike-dependent increases in pump activity, which is affected by dopamine. Understanding how sodium pumps contribute to network regulation and are targeted by neuromodulators, including dopamine, has clinical relevance due to the role of the sodium pump in diseases, including amyotrophic lateral sclerosis, parkinsonism, epilepsy, and hemiplegic migraine.

## Introduction

The Na^+^/K^+^-ATPase (or “sodium pump”) is one of the most abundantly expressed proteins in the nervous system. Sodium pumps are active continuously, using energy derived from ATP, to transport three Na^+^ ions out and two K^+^ ions into cells to maintain appropriate ionic gradients across the cellular membrane (Kaplan, 2002). The unequal nature of this exchange of positive charge enables pumps to contribute to the resting membrane potential (RMP; i.e., the sodium pump is electrogenic). Moreover, activity- and state-dependent changes in sodium pump activity can alter the outward, hyperpolarizing pump current and modify neuronal properties.

Sodium pump currents are commonly increased when Na^+^ ions accumulate intracellularly during prolonged or intense firing. There are four catalytic α-subunit isoforms (α1-α4), with neurons predominantly expressing α1 and α3 (Hieber et al., 1991). The α3 subtype has a lower Na^+^ affinity and is submaximally active in resting neurons, allowing it to act as a sensor for activity-dependent rises in intracellular Na^+^ (Dobretsov and Stimers, 2005; Azarias et al., 2013). The subsequent increase in the activity of α3-containing sodium pumps not only restores intracellular Na^+^ levels but can also generate a membrane hyperpolarization that reduces the excitability of the neuron for tens of seconds.

Activity-dependent sodium pump currents are described in many cell types across diverse species. These include sensory neurons in leech and lamprey (Parker et al., 1996; Scuri et al., 2002), presynaptic and postsynaptic Calyx of Held neurons (Kim et al., 2007), mammalian cerebellar Purkinje fibers (Forrest et al., 2012), CA1 pyramidal neurons (Gulledge et al., 2013), dopaminergic midbrain neurons (Johnson et al., 1992), and suprachiasmatic nucleus neurons (Wang and Huang, 2006). Pump currents play an especially important role in regulating rhythmically active networks, such as the central pattern generators (CPGs) that coordinate locomotion. In *Xenopus* tadpoles, sodium pumps generate a spike-dependent hyperpolarization in spinal neurons that both weakens and terminates swimming, and inhibits future activity for around a minute, acting as a short-term motor memory mechanism linking past to future network activity (Zhang and Sillar, 2012; Zhang et al., 2015). Similarly, *Drosophila* larvae motoneurons generate a pump current that regulates the frequency of crawling locomotor behavior (Pulver and Griffith, 2010).

The role of the sodium pump in the rhythm-generating networks of the mammalian brainstem and spinal cord is less well described. In the brainstem respiratory network, termination of respiratory-related bursts is partly mediated by enhanced pump current, among other Na^+^-dependent outward currents (Krey et al., 2010; Tsuzawa et al., 2015). Within the rat spinal cord, where α3-containing sodium pump expression is high (Watts et al., 1991), blockade of the sodium pump disrupts disinhibited bursting induced by strychnine and bicuculline, causing activity to first become sporadic and then cease altogether (Ballerini et al., 1997). Similar results have been reported in rat spinal cord organotypic slice cultures (Darbon et al., 2003). A recent study characterized the distribution of the α1 and α3 subunits in the mouse spinal cord and found widespread expression of α3 throughout the ventral and dorsal horn (Edwards et al., 2013). However, no previous study has explored the effects of sodium pump manipulation on locomotor-related activity in the mouse, or has characterized an activity-dependent, sodium pump-mediated hyperpolarization in mouse spinal neurons.

Here we show that sodium pump blockade increases the frequency of drug- and sensory-induced locomotor activity in neonatal mice, whereas pump activation has the opposite effects. We also show that the duration of sensory-evoked locomotor bouts is restricted by sodium pump activity and that interepisode interval influences bout duration and burst frequency through a pump-mediated mechanism. Using whole-cell patch-clamp recordings, we identify a spike-dependent sodium pump hyperpolarization in motoneurons and interneurons. This pump potential is abolished in a dose-dependent manner by ouabain, blocked by TTX, mimicked by monensin, and enhanced by dopamine (DA). These results highlight the importance of the sodium pump both as a dynamic regulator of the mammalian locomotor network and as an important spinal target for dopaminergic signaling.

## Materials and Methods

### 

#### Experimental animals

All experimental procedures were conducted in accordance with the United Kingdom Animals (Scientific Procedures) Act, 1986, approved by the Animal Welfare Ethics Committee of the University of St Andrews and conformed to United Kingdom Home Office regulations. Whole spinal cord preparations used for ventral root recordings were obtained from postnatal day 1–4 C57BL/6 mice of either sex. Spinal cord slice preparations used for whole-cell patch-clamp recordings were obtained from postnatal day 2–15 C57BL/6 mice of either sex. Cre-Lox recombination was used to target Pitx2^+^ interneurons by crossing *Pitx2::Cre* mice (Liu et al., 2003) with homozygous *ROSA-loxP-STOP-loxP-tdTomato* fluorescent reporter animals (Madisen et al., 2010). The *Pitx2::Cre: ROSA-loxP-STOP-loxP-tdTomato* animals exhibited fluorescence in Pitx2^+^ cells (Zagoraiou et al., 2009), which were targeted in a subset of single-cell electrophysiology experiments.

Spinal cords were isolated using techniques similar to those described by Jiang et al. (1999). In summary, animals were killed via cervical dislocation, decapitated, and eviscerated. Then, the spinal column, pelvic girdle, and hindlimbs were transferred and pinned, ventral side up, to the bottom of a chamber containing aCSF (equilibrated with 95% oxygen, 5% carbon dioxide, ∼4°C). Under a dissecting microscope, vertebral bodies were carefully removed using forceps and microscissors to reveal the spinal cord. Spinal cord tissue from midcervical to upper sacral segments was then floated out of the spinal canal by snipping spinal roots on both sides and removing further connective tissue. The dorsal and ventral roots were then trimmed on both sides. For ventral root recordings, the preparation was transferred to a recording chamber superfused with oxygenated aCSF (flow rate 8–12 ml/min). The spinal cord was then pinned onto Sylgard resin inside this chamber with the ventral side up, ready for electrophysiological recordings.

For single-cell recordings, P2-P15 mouse spinal cords were isolated as above using dissecting aCSF (equilibrated with 95% oxygen, 5% carbon dioxide, ∼4°C). Spinal cord slices (300 μm thickness) from lumbar segments were obtained using a vibratome (VT1200, Leica) and transferred to recovery aCSF, which was kept at ∼34°C, and continuously bubbled with 95% oxygen, 5% carbon dioxide for at least 1 h. Slices were then transferred to a beaker containing aCSF (equilibrated with 95% oxygen, 5% carbon dioxide at room temperature).

#### Drugs and solutions

The aCSF used for dissections and recordings contained the following (in mm): 127 NaCl, 3 KCl, 1.25 NaH_2_PO_4_, 1 MgCl_2_, 2 CaCl, 26 NaHCO_3_, and 10 glucose. The dissecting aCSF contained the following (in mm): 25 NaCl, 188 sucrose, 1.9 KCl, 1.2 NaH_2_PO_4_, 10 MgSO_4_, 1 CaCl, 26 NaHCO_3_, 25 glucose, and 1.5 kynurenic acid. The recovery aCSF contained the following (in mm): 119 NaCl, 1.9 KCl, 1.2 NaH_2_PO_4_, 10 MgSO_4_, 1 CaCl, 26 NaHCO_3_, 20 glucose, and 1.5 kynurenic acid. Intracellular solution used for single-cell recordings contained the following (in mm): 140 KMeSO_4_, 10 NaCl, 1 CaCl, 10 HEPES, 1 EGTA, 3 Mg-ATP, and 0.4 GTP-Na_2_. All drugs were supplied by Sigma-Aldrich, dissolved in H_2_O and stored in aliquots at −20°C before use.

#### Electrophysiological recordings

##### Ventral root experiments.

**Figure 1.**
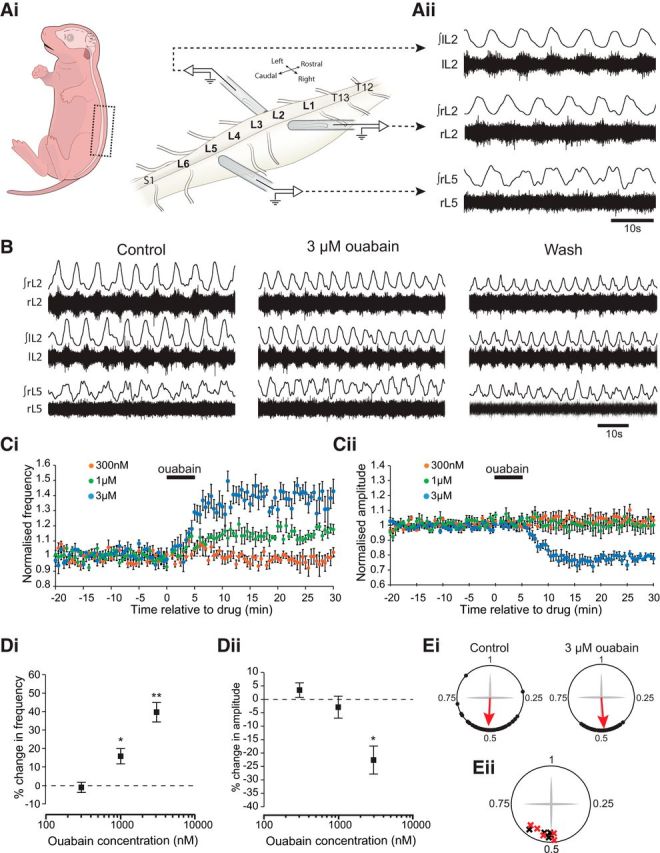
Ouabain increases the frequency and reduces the amplitude of drug-induced locomotor activity in a dose-dependent manner. ***Ai***, Schematic depicting neonatal mouse spinal cord preparation. Glass suction electrodes are attached to the first or second lumbar ventral roots (L_1_, L_2_) on the left and right sides of an isolated spinal cord to record flexor-related activity, and a third electrode is attached to the fifth ventral root (L_5_) to record extensor-related activity. ***Aii***, Raw and rectified/integrated traces showing drug-induced activity on the left and right L_2_ root and the right L_5_ root. ***B***, Equivalent traces showing activity on the left and right L_2_ root and the right L_5_ root, illustrating the effects of 3 μm ouabain. ***Ci***, Time-course plot showing normalized frequency values averaged into 30 s bins. Ouabain causes a concentration-dependent increase in locomotor frequency. ***Cii***, Time-course plot showing normalized amplitude values. Ouabain causes a concentration-dependent decrease in the amplitude of locomotor activity. ***Di***, Mean change in locomotor burst frequency at three different concentrations of ouabain (300 nm, *n* = 7; 1 μm, *n* = 7; 3 μm, *n* = 7). *p < 0.05. **p < 0.01. ***Dii***, Mean change in locomotor burst amplitude at the same concentrations described above. *p < 0.05. ***Ei***, Circular plots illustrating the relative phase of bursts recorded from the left L_2_ ventral root relative to the right L_2_ ventral root. The plots represent 50 bursts recorded in control and 50 bursts recorded in the presence of 3 μm ouabain (taken from the same experiment shown in ***A***,***B***). Data points are clustered around 0.5 in both conditions, illustrating left-right alternation in both conditions (*p* < 0.05). ***Eii***, Circular plot depicting mean left-right values in control (black crosses) and 3 μm ouabain (red crosses) across several experiments (*n* = 5, *p* < 0.05).

For experiments in which locomotor-related activity was induced pharmacologically, glass suction electrodes were attached to the first or second lumbar ventral roots (L_1_, L_2_) on both the left and right side of isolated spinal cords to record left-right alternating, flexor-related activity. In the majority of experiments, a third electrode was also attached to the fifth lumbar ventral root (L_5_) to record extensor-related activity (see Fig. 1*Ai*). NMDA (5 μm), 5-hydroxytryptamine (5-HT; 10 μm), and DA (50 μm) were added to the aCSF to induce rhythmic bursts of locomotor-related ventral root activity that alternated between the left and right sides of the spinal cord and between ipsilateral flexor (L_1_, L_2_) and extensor (L_5_) related ventral roots. For a subset of experiments, only NMDA and 5-HT were used to induce locomotor-related activity, with DA omitted. Consistent with previous studies (e.g., Foster et al., 2014; Sharples et al., 2015), the frequency of bursting was faster and the rhythm less stable in the absence of DA. Drugs used to manipulate sodium pump activity were applied once the observed amplitude, frequency, and duration of rhythmic bursting had stabilized (usually after ∼1 h). For dorsal root stimulation experiments, glass suction electrodes were attached to the first or second lumbar ventral roots (L_1_, L_2_) on both the left and right side of isolated spinal cords, while a stimulating electrode was attached to the third, fourth, or fifth lumbar dorsal root (L_3_, L_4_, or L_5_) on either the left or right side. A series of 40 current pulses (4 Hz) was delivered to the caudal stimulating electrode every 2 min, using a Master-8 pulse generator and iso-flex pulse stimulator (A.M.P.I.), to evoke episodes of locomotor-like activity (as in Whelan et al., 2000). The amplitude of the pulses was set to 50–100 μA. In an initial set of experiments, we found that DA (50 μm) stabilized the sensory-evoked motor output and therefore was added to the aCSF for the remaining dorsal root stimulation experiments. For both drug- and sensory-induced activity, signals were amplified and filtered (30–3000 Hz; Qjin Design) and then acquired at a sampling frequency of 6 kHz using a Digidata 1440A A/D board and AxoScope software (Molecular Devices). Simultaneous online rectification and integration were performed on each raw ventral root signal during the recording.

##### Patch-clamp experiments.

Spinal cord slices were immersed in a recording chamber with aCSF continuously reperfused (50 ml) at a constant rate (1 ml per second). Whole-cell patch-clamp recordings were performed from ventral horn interneurons and motoneurons using glass microelectrodes (2.5–5 mΩ) filled with intracellular solution. Signals were amplified and filtered (4 kHz low-pass Bessel filter) with a MultiClamp 700B amplifier (Molecular Devices) and acquired at ≥10 kHz using a Digidata 1440A A/D board and pClamp software (Molecular Devices). Gigaseals (>2 GΩ) were obtained before the establishment of whole-cell mode and neurons with RMP between −40 mV and −80 mV were used for experiments. All recordings were performed in current-clamp mode. The pump-mediated hyperpolarization was elicited by either applying a 10 s supramaximal continuous depolarizing pulse, or if the neuron exhibited single spike or adaptive spiking behavior, high-frequency stimulation (20–100 Hz) was used. A hyperpolarizing, bias current (≤−50 pA) was sometimes injected into the neuron if it depolarized following drug perfusion. This prevented any significant differences in firing frequency during stimulation, allowing us to fairly compare pump potentials between control and drug conditions. To evaluate input resistance before and after stimulation, we injected 20 pA hyperpolarizing pulses (see Results). All experiments were conducted at room temperature (∼20°C).

#### Data analysis

Extracellular electrophysiological data were first analyzed using Dataview software (version 10.3.0, courtesy of Dr. W. J. Heitler), and all raw data were imported into Excel spreadsheets and analyzed. Statistical analyses were conducted using PASW statistics 21 or Excel. The means of each condition were compared using paired Student's *t* tests or repeated-measures ANOVAs followed by Bonferonni-corrected *t* tests, unless otherwise stated. Results are reported as mean ± SEM. For determination of left-right phase values, we analyzed 50 bursts recorded from the left and right second lumbar root (L_2_)and applied circular statistics. Phase values were calculated by dividing the latency between a consecutive left and right burst by the cycle period on the side of the leading burst (for details, see Kjaerulff and Kiehn, 1996). Phase values for flexor-extensor alternation were calculated using the same method as above but using bursts from the second lumbar root (L_2_) and fifth lumbar root (L_5_) on the same side. Where relevant, the phase values from a sample individual experiment are plotted on a circular plot for each condition, with the mean phase indicated by an arrow and the length of the arrow indicating the strength of coupling. Mean phase values for the full set of experiments are also shown on a circular plot, with crosses indicating mean phase values and the color indicating the condition. Rayleigh's test for uniformity was used to statistically assess whether any of our drug conditions affected left-right or flexor-extensor coupling.

For patch-clamp experiments, data were analyzed using ClampFit software (Molecular Devices) and then exported to Excel spreadsheets for statistical analysis. Prestimulation resting voltage was compared with immediate poststimulation voltage to address the amplitude of the pump AHP. The time between the end of the stimulation and the end of the hyperpolarizing phase was used to calculate the duration of the pump AHP. Because of its slow kinetics (e.g., Zhang and Sillar, 2012), the pump potential was defined as a slow hyperpolarization lasting ≥20 s. Paired Student's *t* tests were used to determine statistically significant changes between control and drug conditions.

#### Histology

##### Immunohistochemistry.

Transverse spinal cord sections were prepared from a *Pitx2::Cre: ROSA-loxP-STOP-loxP-tdTomato* positive mouse (P14) for immunohistochemical staining. Briefly, spinal cord was removed from the animal (as described above for electrophysiological experiments) and fixed immediately in 4% PFA (Sigma-Aldrich) in 0.01 m PBS (Bioline) for 4 h at 4°C, before overnight incubation in 30% sucrose (Sigma-Aldrich). The sample was embedded and frozen in Cryo-M-Bed (Fisher Scientific). Cryosections were prepared at −20°C at 20 μm thickness and mounted onto Superfrost Plus slides (ThermoFisher). Tissue slices were blocked and permeabilized in 3% BSA (Fisher Scientific) and 0.2% Triton X100 (Fisher Scientific) in PBS for 1.5 h at room temperature in a wet chamber. Samples were incubated for 24 h at 4°C in primary antibody solution of mouse anti-α3 (Abcam, ab2826) diluted 1:250 in 1.5% BSA and 0.1% Triton X100 in PBS. After primary antibody incubation, slides were washed 5 times in PBS. Slides were then incubated in FITC-conjugated secondary antibody (Abcam, ab98692) at 1:250 dilution in PBS for 1.5 h in a wet chamber at room temperature. Slides were washed a further 5 times in PBS before DAPI staining at 1:2000 dilution in deionized H_2_O and mounted using Vectashield Hard Set mountant.

##### Fluorescence microscopy.

Fluorescence imaging was performed at 40 × magnification using a Zeiss Axio Imager M2 microscope with Apotome.2. Serial multichannel *Z*-stacks were acquired over 15–25 μm to capture the full slice with *Z*-increments of 0.8 μm. Exposure time for the α3-FITC stain was kept standardized for all images, including primary antibody negative controls (1000 ms), whereas exposure times for the TdTomato expression and DAPI were adjusted in accordance with their fluorescence intensities in the slice. Analysis of positive α3 expression in neurons was determined as green fluorescence rings or numerous focal spots around the cell body.

## Results

### Sodium pump inhibition increases locomotor frequency

The role of the sodium pump within mouse spinal motor networks was first investigated by applying the cardiac glycoside inhibitor of the pump, ouabain, to isolated spinal cord preparations while recording locomotor-related activity from the ventral roots (Fig. 1*A*,*B*). Stable locomotor activity was induced by the bath application of NMDA (5 μm), 5-HT (10 μm), and DA (50 μm) (Fig. 1*Aii*). Because different subtypes of the sodium pump are differentially sensitive to ouabain (Lichtstein and Rosen, 2001), we used a range of concentrations from 300 nm to 30 μm (Fig. 1*C*,*D*). We found no significant effects of 300 nm ouabain (*p* > 0.05, *n* = 7). However, bath application of 1 μm ouabain caused a rapid, clear, and significant increase in the frequency of locomotor bursts of ∼15% (15.8 ± 4.1%, *p* < 0.05, *n* = 7) with no significant change in burst amplitude. A higher concentration of ouabain (3 μm; Fig. 1*B*,*C*) elicited a similar, but more pronounced, increase in locomotor burst frequency of ∼40% (39.5 ± 5.2%, *p* < 0.01, *n* = 7) but also significantly reduced the amplitude of bursts by ∼20% (22.6 ± 4.3%, *p* < 0.05, *n* = 7). Finally, we bath-applied a high dose of ouabain (30 μm), which rapidly increased the frequency (reaching a peak of an ∼200% increase) but then quickly abolished activity altogether (data not shown, *n* = 2). Finally, we assessed whether ouabain affected the phase relationships between alternating left-right and flexor-extensor related activity, which involves reciprocal inhibition between interneuron populations organized as half-centers. At all concentrations tested, ouabain had no effect on the left-right alternating pattern of locomotor bursting, which was found to be significantly clustered around a mean phase value of ∼0.5 in both control (*p* < 0.05), and drug (*p* < 0.05), conditions (Fig. 1*B*,*Ei*,*Eii*). Flexor and extensor alternation was similarly unaffected (Fig. 1*B*). These results demonstrate that decreasing the activity of the sodium pump in the spinal cord accelerates the frequency of drug-induced locomotor bursting.

### Sodium pump activation decreases locomotor frequency

**Figure 2.**
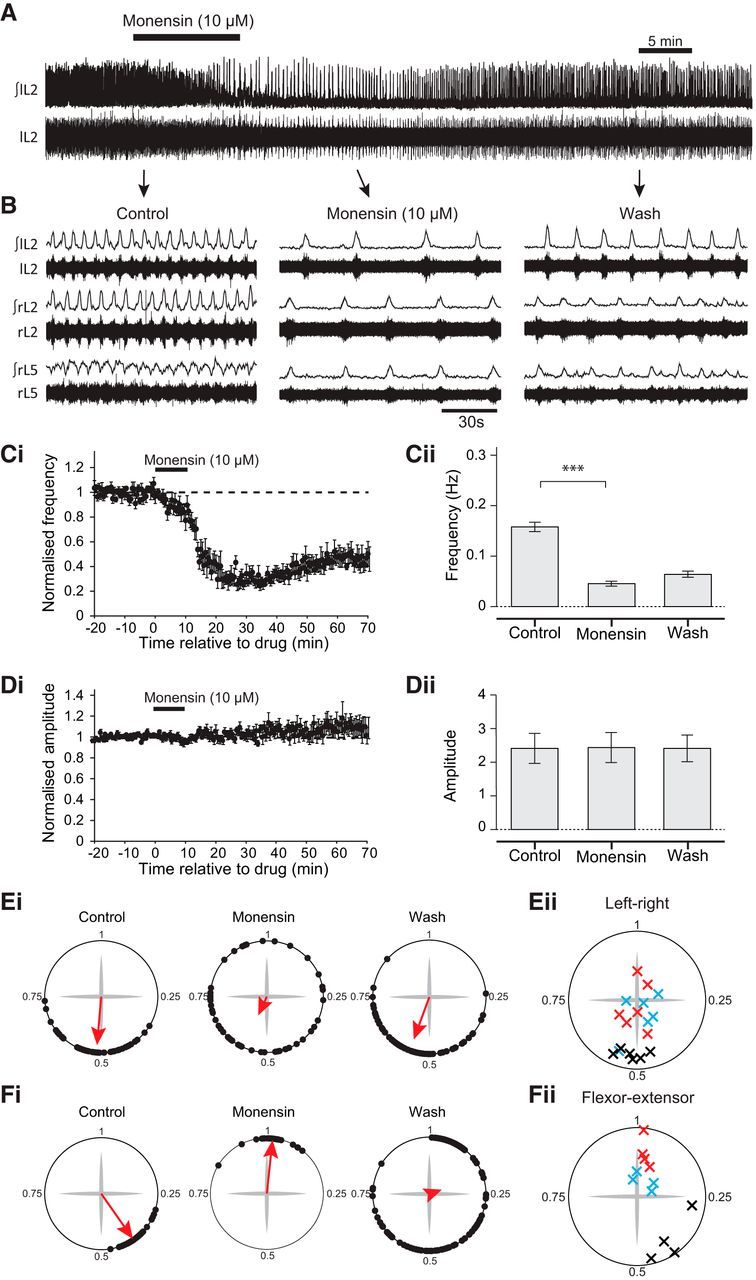
Activation of the sodium pump using the sodium ionophore monensin slows the frequency of drug-induced locomotion and disrupts left-right and flexor-extensor coupling. ***A***, Raw and rectified/integrated recordings from the left L_2_ root, showing an entire experiment to illustrate the effects of 10 μm monensin. ***B***, Raw and rectified/integrated traces showing activity on the left and right L_2_ root and the right L_5_ root, illustrating the effect of the sodium ionophore monensin (10 μm). ***Ci***, Time-course plot showing normalized frequency values averaged into 30 s bins. Monensin (10 μm) causes a decrease in locomotor frequency. ***Cii***, Mean locomotor burst frequency during control, 25 min after the addition of 10 μm monensin, and 45 min after drug washout (*n* = 7). ***Di***, Time-course plot showing normalized amplitude values. Monensin (10 μm) had no significant effect on locomotor burst amplitude (*n* = 7). ***Dii***, Mean locomotor burst amplitude during control, 25 min after the addition of 10 μm monensin, and 45 min after drug washout (*n* = 7). ***Ei***, Circular plots for the experiment shown in ***A*** depicting left-right phase values. Monensin caused a loss of left-right alternation. ***Eii***, Left-right phase values for individual experiments in control (black crosses), monensin (red crosses), and wash (blue crosses) (*n* = 6; 50 bursts for each L_2_ root). ***Fi***, Circular plots for the experiment shown in ***A*** depicting flexor-extensor relationship. Monensin caused flexor and extensor activity to become synchronous. ***Fii***, Flexor-extensor phase values for individual experiments in control (black crosses), monensin (red crosses), and wash (blue crosses) across experiments (*n* = 4). ***p < 0.001.

Next, we wanted to explore the effects of increasing the activity of the sodium pump on drug-induced locomotor activity. There are no known direct pharmacological activators of the sodium pump, but previous studies have used the sodium ionophore monensin to increase intracellular Na^+^ concentration, which has been shown to trigger an increase in sodium pump activation (e.g., Wang et al., 2012; Zhang et al., 2015; Kueh et al., 2016). Bath application of 10 μm monensin to isolated spinal cord preparations led to a significant decrease in locomotor frequency of ∼70%, the opposite effect to ouabain (−70.6 ± 4.3%; Fig. 2*A–C*; *p* < 0.001, *n* = 7). There was no significant effect of monensin on burst amplitude (Fig. 2*D*; *p* > 0.05, *n* = 7). However, we found that monensin disrupted left-right and flexor-extensor coupling (Fig. 2*B*,*E*,*F*). We observed normal left-right alternation in control conditions, with a mean phase value significantly clustered around a value of 0.5 (*p* < 0.001, *n* = 6). In monensin, phase values became uniformly distributed (*p* = 0.2, *n* = 6), indicating a loss of this left-right coupling. We also observed normal flexor-extensor alternation in control (Fig. 2*B*,*F*; *p* < 0.05, *n* = 4). However, in monensin, although significant coupling remained (*p* < 0.01, *n* = 4), the mean values were clustered around a value of 1, indicating flexor-extensor synchrony. These results suggest that activation of the sodium pump in the spinal cord, by raising intracellular sodium, decelerates the frequency of a drug-induced locomotor bursting rhythm, causes a decoupling of the left and right sides of the spinal cord, and synchronizes the normally alternating flexor-extensor relationship.

### DA-mediated modulation of locomotor activity involves effects on the sodium pump

**Figure 3.**
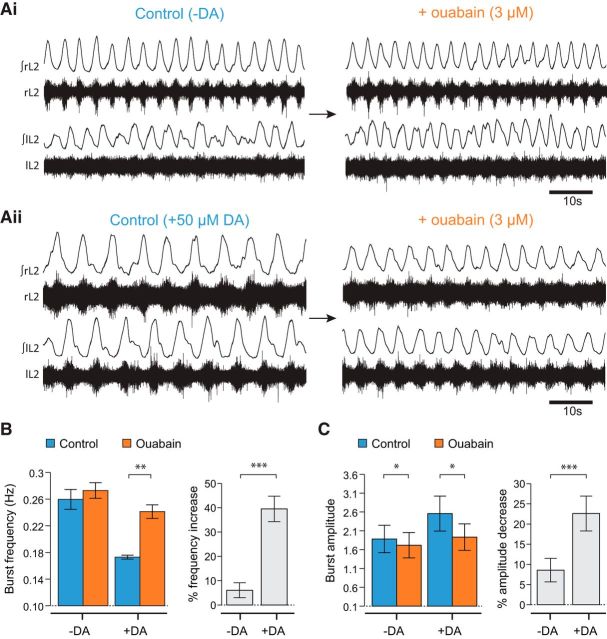
The effects of ouabain on locomotor output are dependent on the presence of DA. ***Ai***, Raw and rectified/integrated traces showing locomotor-like activity in the absence of DA in control and in the presence of 3 μm ouabain. ***Aii***, Raw and rectified/integrated traces showing locomotor activity recorded in the presence of 50 μm DA in control and in the presence of 3 μm ouabain. ***B***, Pooled locomotor burst frequency data. In the absence of DA, 3 μm ouabain had no significant effect on the locomotor burst frequency. However, in the presence of 50 μm DA, 3 μm ouabain caused a clear and significant increase in the frequency of locomotor bursts. ***C***, Pooled locomotor burst amplitude data. 3 μm ouabain had a larger effect on burst amplitude when applied in the presence of DA; however, the change was significant both with and without DA. Overall, these data suggest that the effects of sodium pump blockade are dependent on the presence of DA. *p < 0.05. **p < 0.01. ***p < 0.001.

DA lowers the frequency and increases the amplitude of locomotor bursting induced by NMDA and 5-HT (Sharples et al., 2015), but the mechanisms through which DA acts remain largely unknown. One possibility is that DA modulation could involve the activation of the sodium pump, for which precedents exist (Therien and Blostein, 2000). To test this possibility, we conducted a series of experiments in which ouabain (3 μm) was bath-applied to preparations in which DA was excluded from the combination of drugs used to induce locomotor-related activity. Consistent with previous findings, the locomotor pattern in the absence of DA displayed a higher frequency of bursting with reduced amplitude (Fig. 3*Ai*), reminiscent of activity in the presence of a sodium pump blocker. Importantly, in the absence of DA, bath-applied ouabain no longer had a significant effect on burst frequency (*p* > 0.05, *n* = 7; Fig. 3*A*,*B*). Without DA present, ouabain still significantly decreased burst amplitude (*p* < 0.05, *n* = 7; Fig. 3*A*,*C*); however, there was a clear and significant difference between the magnitude of the amplitude effect compared with experiments in the presence of DA (22.6 ± 11.4% DA vs 8.6 ± 7.7% without DA, *p* < 0.001). These results demonstrate that the effects of ouabain are substantially dependent on the presence of DA, suggesting that DA may be acting in the spinal cord as a neuromodulator partly through mechanisms involving the sodium pump.

### Sodium pump activity modulates sensory-induced locomotor bouts

**Figure 4.**
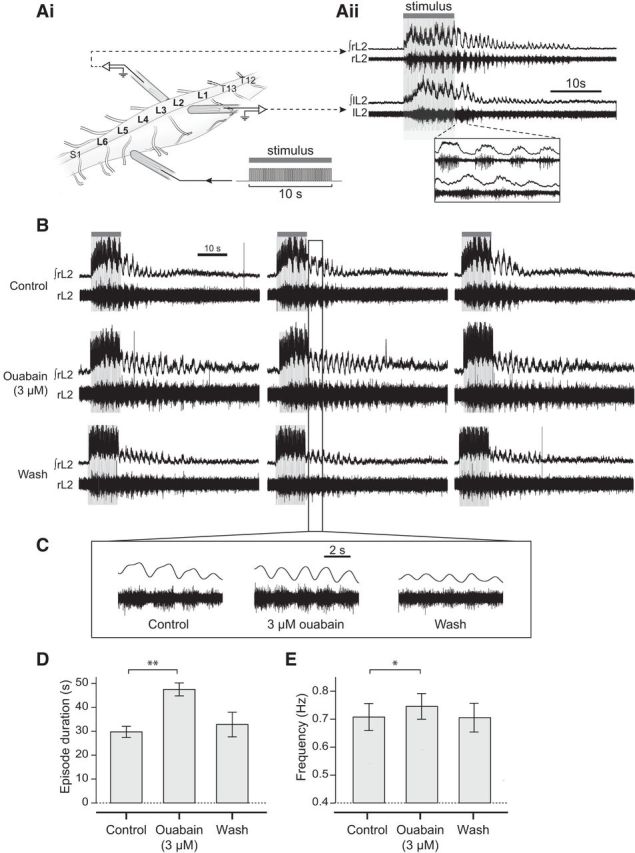
The sodium pump blocker ouabain increases the duration and frequency of sensory-evoked locomotor activity. ***Ai***, Schematic illustrating the experimental setup. Glass suction electrodes were attached to the first or second lumbar ventral roots (L_1_, L_2_) on the left and right side of an isolated spinal cord to record flexor-related activity. A third electrode was also attached to the fifth dorsal root (L_5_) on either the left or right side and used to deliver a series of current pulses to initiate locomotion (see Materials and Methods). ***Aii***, An example of the locomotor output recorded in response to the stimulus delivered to the dorsal root. Inset, Left-right alternation. ***B***, Raw and rectified/integrated traces showing 3 consecutive stimulus-evoked episodes of locomotor activity in control, in the presence of 3 μm ouabain, and following washout of the drug. ***C***, Expanded raw traces showing several locomotor bursts in each condition to illustrate the effect of ouabain on locomotor burst frequency. ***D***, Pooled data showing the effects of ouabain on sensory-evoked episode duration (*n* = 7). ***E***, Pooled data showing the effect of ouabain on locomotor burst frequency (*n* = 7). *p < 0.05. **p < 0.01.

**Figure 5.**
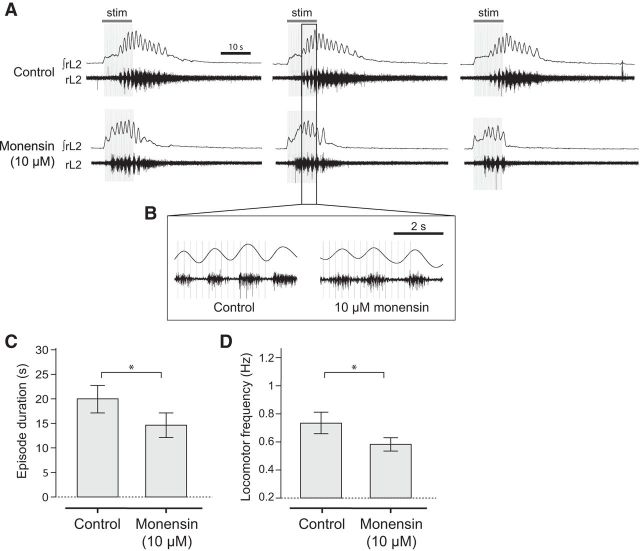
Activation of the sodium pump using the sodium ionophore monensin decreases both the duration and frequency of evoked locomotor activity. ***A***, Raw and rectified/integrated traces showing three sensory-evoked episodes of locomotor activity in control and in the presence of 10 μm monensin. ***B***, Expanded raw traces showing several locomotor bursts in each condition to illustrate the effect of monensin on locomotor burst frequency. ***C***, Pooled data showing the effects of monensin on sensory-evoked episode duration (*n* = 7). ***D***, Pooled data showing the effect of monensin on locomotor burst frequency (*n* = 7). *p < 0.05.

Next, we wanted to explore whether the effects of sodium pump manipulation also occur using an alternative and potentially more physiological method for generating motor output. Therefore, we used dorsal root stimulation to induce brief episodes (∼20 s long) of locomotor-like activity. Given that sodium pumps have a greater influence on locomotor network activity when DA is present, the following dorsal root stimulation experiments were all performed with 50 μm DA included in the aCSF. Ouabain (3 μm) significantly extended the duration of sensory-evoked locomotor episodes by ∼70% (66.7 ± 15.8% Fig. 4*B*,*D*; *p* < 0.01, *n* = 7) and elicited a small but significant increase in the frequency of locomotor activity of ∼6% (5.9 ± 1.7%; Fig. 4*B*,*C*,*E*; *p* < 0.05, *n* = 7). These data suggest that the duration and frequency of sensory-evoked locomotor episodes are somehow restricted by a pump-dependent mechanism. If so, then increased pump activity would be expected to decrease episode durations and slow the frequency. As predicted, monensin (10 μm) reduced the duration of sensory-evoked locomotor episodes by ∼24% (23.7 ± 4.5%; Fig. 5*A–C*; *p* < 0.05, *n* = 7), before eventually abolishing activity altogether. In addition, the frequency of locomotor bursts within the shortened episodes of activity was significantly slowed by ∼20% (18.8 ± 5.2%; Fig. 5*B*,*D*; *p* < 0.05, *n* = 7); opposite to the effects of ouabain. Collectively, these results illustrate that changes in sodium pump activity in the spinal cord modulate the duration and frequency of sensory-induced bouts of locomotor activity.

### Effects of interepisode interval on sensory-evoked locomotor activity

In other preparations, including *Xenopus* tadpoles and *Drosophila* larvae, the amplitude and duration of a pump current generated by CPG neurons during locomotion encode information about locomotor network output in an activity-dependent manner. This allows the network to retain a short-term memory of recent activity, enabling future network output to be influenced in an interval-dependent fashion. In *Xenopus* tadpoles, this “motor memory” can last up to 1 min; if swimming is evoked within this minute, the unrecovered pump hyperpolarization inhibits spinal neurons, causing the second episode to be slower, weaker, and shorter in duration (Zhang and Sillar, 2012; Zhang et al., 2015).

**Figure 6.**
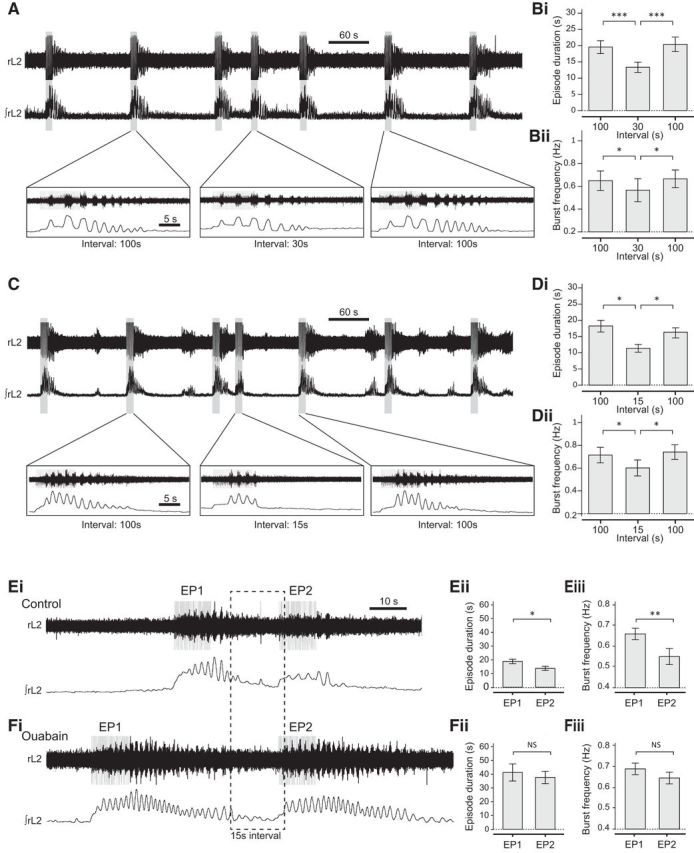
The sodium pump mediates the interval relationship between evoked episodes of fictive locomotor activity. ***A***, Raw and rectified/integrated traces showing a series of sensory-evoked episodes, with expanded traces below. The first three episodes are separated by a 2 min stimulation interval. Between the third and fourth episodes shown, the interval is set to 30 s, before returning to a 2 min stimulation interval. ***Bi***, Episodes evoked after a 30 s interval were significantly shorter in duration compared with episodes evoked after a 2 min interval. ***Bii***, Episodes evoked after a 30 s interval had significantly slower frequency compared with episodes evoked after a 2 min interval. ***C***, Raw and rectified/integrated traces showing a series of sensory-evoked episodes. Inset, Expanded traces. The first three episodes are separated by a 2 min stimulation interval. Between the third and fourth episode shown, the interval is set to 15 s. ***Di***, Episodes evoked after a 15 s interval were significantly shorter in duration compared with episodes evoked after a 2 min interval. ***Dii***, Episodes evoked after a 15 s interval had significantly slower frequency compared with episodes evoked after a 2 min interval. ***Ei***, An example of two evoked locomotor episodes separated by a 15 s interval, before the application of ouabain. Similar to the results shown in ***D***, both episode duration (***Eii***, *p* < 0.01, *n* = 5) and burst frequency (***Eiii***, *p* < 0.05, *n* = 5) were significantly reduced in episode 2 (EP2) compared with episode 1 (EP1). ***Fi***, An example of two evoked episodes from the same experiment as ***Ei*** in the presence of 3 μm ouabain but still separated by a 15 s interval. In the presence of ouabain, there was no significant difference in episode duration (***Fii***, *p* > 0.05, *n* = 5) or burst frequency (***Fiii***, *p* > 0.05, *n* = 5) between EP1 and EP2. *p < 0.05. **p > 0.01. ***p < 0.001.

To test whether an activity-dependent pump current may play a similar role in mice, we manipulated the interval between sensory-evoked episodes of locomotor-like activity using a protocol in which locomotor episodes were first evoked with a 2 min separation. After at least three consistently similar control episodes, we then shortened the interval to either 30 or 15 s. Compared with episodes evoked following a 2 min interval, episodes were significantly shorter in duration when following either a 30 s interval (−31.4 ± 5.1%; Fig. 6*A*,*Bi*; *p* < 0.001, *n* = 7) or a 15 s interval (−36.2 ± 4.9%; Fig. 6*C*,*Di*; *p* < 0.05, *n* = 7). Moreover, the locomotor burst frequency was significantly slower following either a 30 s (−16.1 ± 3.9%; Fig. 6*A*,*Bii*, *p* < 0.05; *n* = 7) or a 15 s interval (−16.4 ± 4.5%; Fig. 6*C*,*Dii*, *p* < 0.05; *n* = 7). These results suggest that the locomotor network encodes information about its own previous output and that this information is retained for at least 30 s. Locomotor activity evoked within this period is inhibited in a manner reminiscent of increased sodium pump activity.

To examine whether the sodium pump contributes to this interval effect, we tested whether ouabain (3 μm) altered the relationship between interepisode interval and intraepisode parameters. Following blockade of the sodium pump, episodes separated by a 15 s interval were not only longer and higher in frequency compared with episodes evoked in control conditions (Fig. 6*Fi*, same result as Fig. 4), but unlike in control conditions (Fig. 6*C*,*D*), the second episode of a pair was no longer shorter (Fig. 6*Fi*,*Fii*, *p* > 0.05, *n* = 5) or slower (Fig. 6*Fi*,*Fiii*, *p* > 0.05, *n* = 5) compared with the first episode. These data suggest that feedforward control of successive bouts of locomotor-related activity involves activity-dependent enhancement of sodium pump activity.

### A sodium pump-mediated afterhyperpolarization (AHP) in spinal neurons

**Figure 7.**
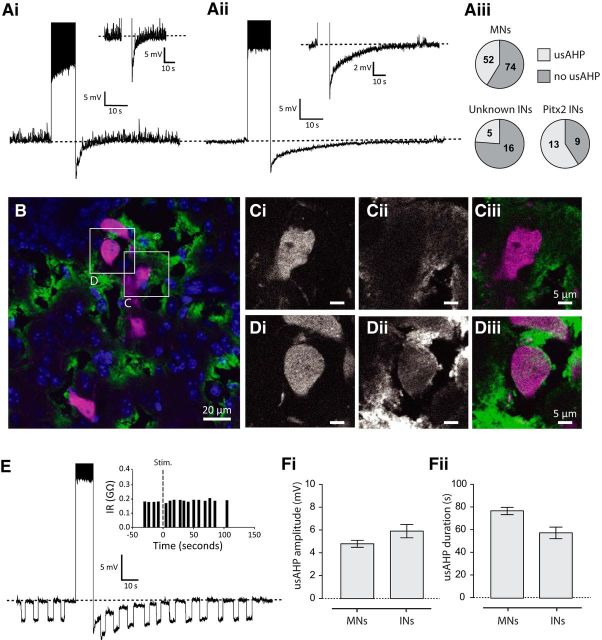
A spike-dependent usAHP in motoneurons and interneurons and α3 sodium pump expression. ***Ai***, An example of a motoneuron displaying a short duration AHP that lasts only ∼5 s. ***Aii***, An example of an usAHP in a motoneuron in response to a high-frequency train of action potentials, which lasts ∼60 s. ***Aiii***, Summary of the proportion of motoneurons and interneurons displaying a usAHP. ***B–D***, Immunostaining for α3 subunit expression in Pitx2-tdTomato positive spinal cord slices. ***B***, Average *Z*-projection (from 4 μm in depth) of α3 (green), Pitx2-tdTomato (magenta), and DAPI (blue). ***Ci–Ciii***, Negative staining around a Pitx2^+^ interneuron. ***Di–Diii***, Positive staining around a Pitx2^+^ interneuron. ***E***, Responses to short duration current pulses in a motoneuron before and after the induction of a usAHP. Inset, Measurements of conductances before and after stimulation (“stim.”). ***Fi***, Summary of the amplitude of the usAHP in motoneurons and interneurons. ***Fii***, A summary of the duration of the usAHP in motoneurons and interneurons.

The preceding ventral root recordings suggest that sodium pumps play an important role in shaping locomotor output, but what are the effects, if any, at the cellular level? To address this question, we next explored how sodium pump activity affects individual CPG neurons using whole-cell patch-clamp recordings from spinal cord slices. Sodium pump activity can modify neuronal firing via a tonic contribution to the RMP or via a dynamic, transient, activity-dependent AHP. To test for the presence of a dynamic sodium pump hyperpolarization, we induced repetitive spiking in cells in one of two ways: either by applying a large, continuous depolarizing current pulse (10 s duration); or, if a cell showed pronounced spike adaptation, using a 10 s train of short duration (10 ms) depolarizing current pulses. We observed a range of different poststimulation responses. In some cells, we observed no post-tetanic change in membrane potential. In some motoneurons (62 of 126 cells) and some unidentified ventral horn interneurons (16 of 21 cells), we observed a short duration hyperpolarization, with the membrane potential returning to baseline after ∼5–10 s (Fig. 7*Ai*). However, in a large proportion of motoneurons (52 of 126 cells) and a smaller proportion of interneurons (5 of 21 cells), we also observed a prominent, long duration AHP, ∼5 mV in amplitude (4.80 ± 0.3 mV for motoneurons, *n* = 52; 5.9 ± 0.6 mV for interneurons, *n* = 5; Fig. 7*Aii*), where the membrane potential gradually returned to baseline over a period of ∼1 min (76.8 ± 3.2 s for motoneurons, *n* = 52; 57.4 ± 5.2 s for interneurons, *n* = 5). Because of the overlap between short and long duration AHPs, we defined the long AHP as lasting >20 s. The amplitude and duration of this long, post-activity AHP are reminiscent of the minute-long AHP generated by sodium pump activity in *Xenopus* spinal neurons, which has been termed the “ultra-slow afterhyperpolarization (usAHP)” (Zhang and Sillar, 2012), and so we adopt this terminology here in mammalian spinal neurons. We found no significant difference in RMP between neurons with and without a usAHP (with usAHP: −62.78 ± 1.37 mV; without usAHP: 65.87 ± 1.14 mV, *p* > 0.05). Whereas a large proportion of motoneurons (∼40%) displayed a usAHP, it was found in only ∼25% of unidentified ventral horn interneurons, suggesting more restricted expression of the usAHP in specific interneuron subtypes. One interneuron subtype known to contribute to spinal locomotor control and which exhibits activity phase-locked to motoneuron output are Pitx2^+^ interneurons (Zagoraiou et al., 2009). We therefore hypothesized that a higher proportion of this cell type might display a usAHP. Using spinal cord slices from *Pitx2::Cre: ROSA-loxP-STOP-loxP-tdTomato* mice, we found that more than half of Pitx2^+^ interneurons (13 of 22 cells) displayed a usAHP.

Previous experiments have shown that the usAHP is mediated by the sodium pumps containing the α3 subunit (Azarias et al., 2013), which has a heterogeneous distribution in the mouse spinal cord (Edwards et al., 2013), matching our physiological data for usAHP expression. Using immunohistochemistry, we confirmed this finding by Edwards et al. (2013) and found that α3 expression was widespread across the dorsal and ventral horn, but restricted to pockets of unidentified interneurons and motoneurons (Fig. 7*B*). To more carefully quantify the proportion of α3-expressing neurons in a specific identified interneuron population, we measured the number of Pitx2^+^ interneurons displaying α3 staining. In transverse spinal cord slices from a *Pitx2::Cre: ROSA-loxP-STOP-loxP-tdTomato* positive mouse, 76% (31 of 41 cells) of Pitx2^+^ interneurons showed positive staining for the α3 subunit, with the majority of cells showing clear expression around the soma membrane and the axon hillock (e.g., Fig. 7*D*). Thus, the heterogeneous nature of usAHP physiology is partnered by a heterogeneous expression of the α3-containing sodium pump.

**Figure 8.**
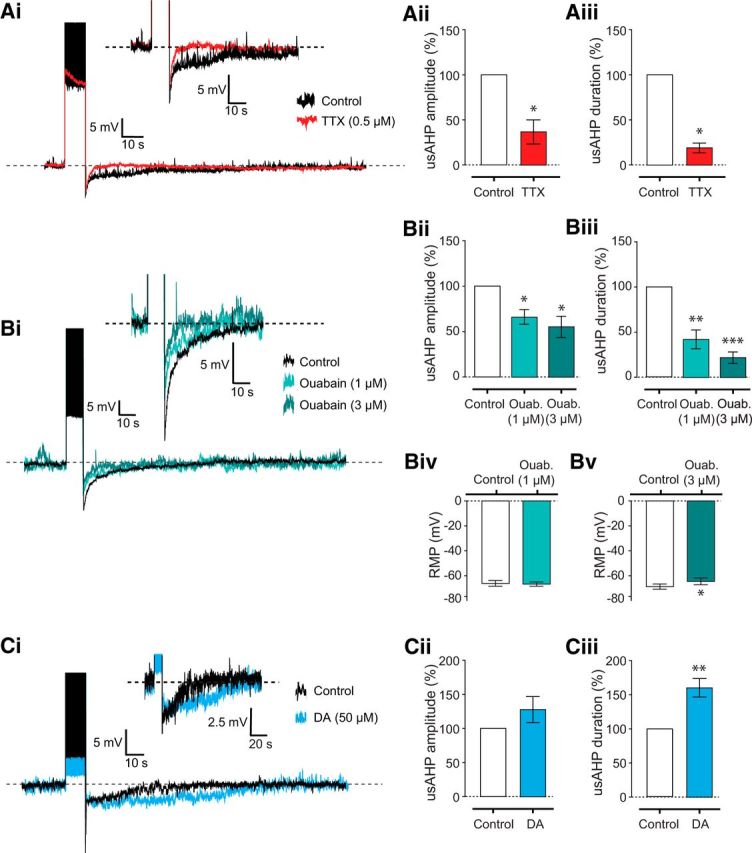
The usAHP in motoneurons is blocked by TTX and ouabain but enhanced by DA. ***Ai***, The sodium channel blocker TTX (0.5 μm) reduces both the amplitude (***Aii***) and duration (***Aiii***) of the usAHP (*n* = 4). ***Bi***, The sodium pump blocker ouabain reduced the usAHP amplitude (***Bii***) and duration (***Biii***) in a dose-dependent manner (1 μm: *n* = 7; 3 μm: *n* = 8). ***Biv***, Ouabain applied at 1 μm had no significant effect on the RMP of neurons (*n* = 7). ***Bv***, Ouabain applied at 3 μm caused a small but significant depolarization of neurons (*n* = 8). ***Ci***, Bath application of 50 μm DA increased the duration (***Ciii***), but not the amplitude (***Cii***), of the usAHP (*n* = 9). *p < 0.05. **p < 0.01. ***p < 0.001.

Next, we wanted to test pharmacologically whether the usAHP is indeed mediated by increased sodium pump activation. Sodium pump AHPs produce no measurable change in membrane conductance (Pulver and Griffith, 2010; Zhang and Sillar, 2012); therefore, we injected hyperpolarizing current pulses to measure the input resistance of the cell before, during, and after the usAHP. We found no significant change in input resistance (Fig. 7*E*). Sodium pump AHPs are also spike-dependent and are therefore abolished when fast sodium channels are blocked. The application of 0.5 μm TTX irreversibly blocked sodium spikes and abolished the usAHP (*p* < 0.05, *n* = 4; Fig. 8*A*), although the short duration hyperpolarization (described above) remained. Because the short duration hyperpolarization was resistant to both TTX and ouabain, we suggest that it is likely to be a Ca^2+^-dependent K^+^ current (K_Ca_, SK), which is known to be expressed in mammalian motoneurons (Miles et al., 2005). Finally, to more directly confirm that the usAHP is produced by sodium pump activity, we applied ouabain (1–3 μm), which decreased both the usAHP amplitude (1 μm: −33.91 ± 8.09%, *n* = 7, *p* < 0.05; 3 μm: −43.91 ± 9.88%, *n* = 8, *p* < 0.05) and duration (1 μm: −59.15 ± 10.66%, *n* = 7, *p* < 0.01; 3 μm: −77.39 ± 5.95%, *n* = 8, *p* < 0.001) in a dose-dependent manner (Fig. 8*B*). We also assessed the effects of these concentrations of ouabain on the RMP. Importantly, although we observed a small, but significant, depolarization in the presence of 3 μm ouabain (*p* < 0.05, *n* = 8; Fig. 8*Bv*), there was no change in membrane potential caused by 1 μm ouabain (*p* > 0.05, *n* = 7; Fig. 8*Biv*); even though this concentration significantly decreased the duration and amplitude of the dynamic usAHP (Fig. 8*Bi–Biii*). Overall, these data demonstrate that a proportion of both motoneurons and ventral horn interneurons, including Pitx2^+^ interneurons, exhibit a spike-dependent, minute-long usAHP. Given that this usAHP is sensitive to both ouabain and TTX and occurs without changes in membrane conductance, we conclude it is most likely mediated by a dynamic, activity-dependent increase in sodium pump activity.

### Modulation of the motoneuron usAHP by DA

The facilitatory effects of 3 μm ouabain on locomotor burst frequency were dependent on the presence of 50 μm DA (Fig. 3), suggesting that DA may affect sodium pump activity in spinal neurons. We therefore tested the effects of 50 μm DA on the usAHP. DA initially depolarized cells and increased spiking (data not shown), so we applied a direct holding current to return the RMP back to control levels. When we repeated the protocol to induce repetitive spiking in the presence of DA, the duration of the usAHP was significantly increased by ∼60% (59.91 ± 13.96%, *n* = 9, *p* < 0.01; Fig. 8*C*). DA had no significant effect on the peak amplitude of the usAHP immediately after cessation of spiking (*n* = 9, *p* > 0.05); however, such measurements were likely complicated by the presence in some cells of short duration, non–pump-related AHPs (Fig. 8*Ai*). These data demonstrate that in motoneurons expressing a pump-mediated outward, hyperpolarizing potential, DA increases the duration of this usAHP.

### Monensin converts a dynamic usAHP into a tonic hyperpolarization

**Figure 9.**
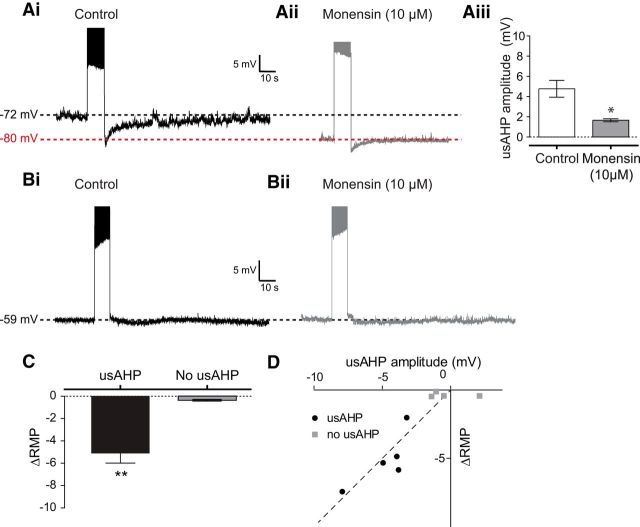
Increasing intracellular sodium using monensin converts a dynamic usAHP into a tonic hyperpolarization. ***A***, The effect of monensin (10 μm) on a motoneuron displaying a usAHP. ***Ai***, Motoneuron displaying a usAHP. ***Aii***, The same cell in the presence of monensin, which caused the membrane potential to hyperpolarize and the usAHP to be abolished. ***Aiii***, Monensin significantly reduced the amplitude of the usAHP (*n* = 5). ***B***, Monensin has no effect on the membrane potential of neurons that lack the usAHP. ***Bi***, Motoneuron exhibiting no usAHP. ***Bii***, The same cell in the presence of monensin, which did not cause a hyperpolarization. ***C***, In motoneurons displaying a usAHP, monensin caused a significant hyperpolarization of the resting membrane potential (*n* = 5), whereas there was no significant change in cells lacking a usAHP (*n* = 4). ***D***, For neurons that did express a usAHP, the size of the hyperpolarization induced by monensin was proportional to the size of the usAHP displayed in control. *p < 0.05. **p < 0.01.

We finally wanted to test whether the usAHP could be enhanced by increasing intracellular sodium using monensin as a proxy for intense network activity. For motoneurons that displayed a large usAHP in control conditions (e.g., Fig. 9*Ai*), the first and most obvious effect of monensin (10 μm) was a membrane hyperpolarization (Fig. 9*A*,*C*; *n* = 5, *p* < 0.01), which was concurrent with a loss of the dynamic usAHP in response to high-frequency spiking (Fig. 9*Aii*,*Aiii*; *n* = 5, *p* < 0.05). The amplitude of this hyperpolarization varied in proportion to the usAHP amplitude observed during control conditions (Fig. 9*D*), strongly suggesting that increased pump activity is mediating the change in membrane potential. Indeed, for neurons that showed no usAHP (e.g., Fig. 9*Bi*), there was no effect of monensin on membrane potential (*n* = 4; Fig. 9*B*,*C*; *p* > 0.05). These data suggest that monensin mimics high activity by increasing intracellular sodium, which in turn activates the sodium pump and generates a hyperpolarization. Unlike sodium influx induced by intense spiking, the pumps are presumably unable to fully counteract the monensin-induced sodium influx and so pump activity remains high; hence, further sodium influx driven by spiking no longer generates a usAHP. Overall, monensin appears to convert a dynamic pump potential into a tonic hyperpolarization by maximizing pump activity.

## Discussion

We show that manipulating the sodium pump using low ouabain concentrations (to block α3-containing pumps) and monensin (to activate the pumps) regulates locomotor activity generated by the mouse spinal cord. We define a sodium spike-dependent usAHP in spinal neurons mediated by dynamic increases in sodium pump activity and enhanced by the neuromodulator DA.

Using two different stimulation methods (pharmacological and sensory), we found that sodium pump blockade increased the frequency of rhythmic locomotor-like activity, whereas pump activation had the opposite effect. Similar effects of ouabain have been reported for the leech heartbeat CPG (Tobin and Calabrese, 2005; Kueh et al., 2016), the rat respiratory CPG (Tsuzawa et al., 2015), and dopaminergic midbrain networks in the rat (Johnson et al., 1992). Ouabain's effects on rhythm frequency could be due to the removal of a tonic, negative contribution of the pump to the RMP, thereby depolarizing specific neurons involved in rhythm generation. However, in many systems, the contribution of the α3-containing sodium pump to the maintenance of RMP is both negligible and slow such that low concentrations of ouabain that selectively block α3, but not α1, have little effect on this component (e.g., Pulver and Griffith, 2010; Zhang and Sillar, 2012). Indeed, we found that 1 μm ouabain, a concentration expected to affect α3, but not α1 (Dobretsov and Stimers, 2005), did not affect the RMP. Moreover, the RMP was similar in neurons with and without a usAHP. The effects of ouabain on locomotor frequency may therefore instead involve the block of a dynamic, activity-dependent pump hyperpolarization (the usAHP). In rhythmically active midbrain dopaminergic neurons (Johnson et al., 1992) and in cultured rat spinal neurons (Darbon et al., 2003), each recurring burst is terminated by a pump-mediated hyperpolarization of several millivolts. Blocking the sodium pump accelerates the rhythm by abolishing this intrinsic interburst delay. Similar effects occur in *Xenopus* tadpole swimming, *Drosophila* larva crawling, and, as shown here, neonatal mouse locomotion. We observed a decrease in burst amplitude in 3 μm ouabain, but not with ≤1 μm, suggesting that these amplitude changes may relate to actions on the tonic, rather than dynamic, sodium pump potential.

Raising intracellular Na^+^ with monensin exerted opposite effects to ouabain on the rhythm, consistent with the activation of a Na^+^-dependent hyperpolarizing dynamic pump potential, which is supported by our patch-clamp recordings. In effect, monensin acts as a proxy for intense, sustained neuronal firing and, rather than depolarizing neurons, drives their membrane potential toward the most hyperpolarized levels attained by the usAHP. Monensin also affected rhythm coordination, disrupting left-right alternation and synchronizing flexor-extensor phase values, presumably through suppression of the activity of interneurons mediating half-center alternation.

The usAHP was present only in a subset of motoneurons and interneurons, matching the heterogeneous usAHP distribution both between and within spinal neuron subtypes documented previously (Darbon et al., 2003; Pulver and Griffith, 2010; Zhang and Sillar, 2012). Given that activity-dependent pump-mediated AHPs are thought to involve increased activation of α3-containing sodium pumps, one possible explanation for this nonuniform physiology is that only cells expressing a dynamic usAHP possess the α3 subunit. Consistent with previous findings (Edwards et al., 2013), we report variable expression of α3 among subpopulations of spinal neurons. Interestingly, approximately three-fourths of Pitx2^+^ interneurons expressed α3, similar to the proportion displaying a usAHP. This suggests that the usAHP is not a generic neuronal phenomenon, but rather a carefully orchestrated feature of rhythmic networks whose distribution is likely correlated with α3 isoform expression. We characterized usAHP expression in the relatively small population of Pitx2^+^ interneurons clustered around the central canal, but it will also be important in the future to identify the other unknown interneuron subtypes expressing a usAHP and to establish the impact of the usAHP upon their contribution to rhythm generation. Given that young animals were used in this study, it will also be interesting to investigate developmental changes in α3 expression and usAHP distribution.

Sodium pump dysfunction is associated with a range of disorders, with three neurological diseases linked to missense mutations in the *ATP1A3* gene encoding the α3 subunit: alternating hemiplegia of childhood (Heinzen et al., 2012), rapid-onset dystonia parkinsonism (de Carvalho Aguiar et al., 2004), and CAPOS syndrome (cerebellar ataxia, areflexia, pes cavus, optic atrophy, and sensorineural hearing loss) (Demos et al., 2014). Moreover, α3 dysfunction has recently been linked to ALS (Ellis et al., 2003; Ruegsegger et al., 2016), epilepsy (Krishnan et al., 2015), and bipolar disorder (Kirshenbaum et al., 2012). The mechanisms are yet to be fully elucidated but may involve a shared inability to respond dynamically and homeostatically to activity-induced rises in intracellular sodium. Recently, several α3-mutant mouse lines have also been established to explore the effects of reduced α3-containing sodium pump activity *in vivo* (Moseley et al., 2007; DeAndrade et al., 2011; Kirshenbaum et al., 2011; Ikeda et al., 2013; Sugimoto et al., 2014; Hunanyan et al., 2015). In all lines, mice show clear motor abnormalities, including hyperambulation (longer and faster bouts of locomotion), abnormal stride lengths, and intermittent dystonia, which may relate to the role of spinal sodium pumps described in this study.

In numerous systems, the sodium pump-mediated usAHP mediates a feedforward control mechanism that influences future activity (Pulver and Griffith, 2010; Zhang and Sillar, 2012; Zhang et al., 2015). Similarly, when we decreased the interval between sensory-evoked locomotor episodes to within the timescale of the usAHP (<1 min), the subsequent locomotor episode became significantly shorter and slower. Ouabain disrupted this relationship, strongly suggesting that changes in sodium pump activity track ongoing locomotor activity and provide a form of short-term motor memory that could function to prevent damage from overexertion. Indeed, increasing evidence suggests that fatigue involves not only peripheral mechanisms (limb muscles), but also central (neuronal) mechanisms, such as reduced motoneuron drive (Meeusen et al., 2006; Ranieri and Di Lazzaro, 2012). There is evidence also in humans that increased sodium pump activity contributes to motor axon hypoexcitability following intense motor activity (Kiernan et al., 2004).

We show that the effects of ouabain on locomotor frequency depended on the presence of DA and that DA significantly enhanced the usAHP in spinal neurons. DA initially depolarized motoneurons, which has previously been shown to be due to the inhibition of resting, leak potassium currents (Han et al., 2007). When this depolarization was negated with current injection, the usAHP was still enhanced. DA is a well-established modulator of locomotor circuits (Miles and Sillar, 2011; Sharples et al., 2014) and is released into the spinal cord during stepping activity in mammals (Gerin and Privat, 1998). However, this may be the first evidence that DA's effects on a spinal motor circuit may involve changes in sodium pump activity. Interestingly, the usAHP was present even in the absence of DA, yet ouabain only significantly affected rhythm frequency when DA was present. One possibility is that DA may reveal a usAHP in critical subpopulations of neurons that do not normally display the usAHP. Alternatively, the usAHP may need to be of sufficient amplitude and/or duration in neurons to exert a significant effect at the level of the whole network.

There are a number of mechanisms through which DA may be acting to mediate changes in the usAHP. One possibility is that DA is acting indirectly on the sodium pump, for example, by enhancing persistent or transient sodium currents, which in turn modify sodium pump activity. Another possibility is that DA may enhance the release of a secondary neuromodulator (so called “metamodulation”), which in turn affects sodium pump activity. Alternatively, there is considerable evidence that DA can act directly on the sodium pump (Zhang et al., 2013). In striatal neurons, D2-like receptor activation stimulates sodium pumps by inhibiting PKA, thus dephosphorylating the α3 subunit (Bertorello et al., 1990; Wu et al., 2007). Other studies have also shown that changes in PKA activity can modulate the ability of a neuron to respond to changes in intracellular sodium through phosphorylation/dephosphorylation of α3 (Azarias et al., 2013). Neuronal sodium pumps can even form complexes with D1/D2 receptors for direct reciprocal modulation (Hazelwood et al., 2008). The detailed mechanisms of DA's action as a pump modulator within spinal motor circuits remain to be determined.

Beyond DA, the sodium pump is a well-established target for a range of modulators (Therien and Blostein, 2000), including those affecting sensory and motor systems. For example, nitric oxide is a known modulator of locomotor circuits (McLean and Sillar, 2004; Foster et al., 2014) and has been shown to decrease sodium pump activity in mammalian spinal neurons (Ellis et al., 2003). In the leech heartbeat CPG, the neuropeptide myomodulin inhibits pump activity to accelerate the rhythm (Tobin and Calabrese, 2005), while serotonin decreases the amplitude of a pump-dependent AHP in tactile sensory neurons (Catarsi et al., 1993). It will be interesting in future studies, therefore, to explore whether the effects of known neuromodulators of mammalian spinal circuits involve interactions with the sodium pump.

Na^+^/K^+^ exchange pumps are ubiquitously distributed, abundantly expressed, and phylogenetically conserved proteins that are often viewed as molecular automata engaged exclusively in the maintenance of ionic distributions across cell membranes. However, as we show here, they respond dynamically to changes in intracellular sodium that accompany intense neuronal firing. This capacity endows networks of the mammalian spinal cord with a homeostatic control mechanism that shapes motor output in an activity-dependent manner. Moreover, our data reveal a novel link between DA signaling, sodium pump activity, and locomotor output, a finding that is relevant to diseases of the motor system in which both the pumps and DA have been implicated.
